# Histoplasmosis in the nasal septum without pulmonary involvement in a patient with acquired immunodeficiency syndrome: case report and literature review

**DOI:** 10.1590/S1516-31802010000400012

**Published:** 2010-07-01

**Authors:** Fernando Oikawa, Daniela Carvalho, Nilce Mitiko Matsuda, Alice Tatsuko Yamada

**Affiliations:** I MD. Resident physician, Hospital Municipal de Campo Limpo "Dr. Fernando Mauro Pires da Rocha", São Paulo, SP, Brazil.; II MD, PhD. Research associate, Department of Surgery and Anatomy, Faculdade de Medicina de Ribeirão Preto, Universidade de São Paulo (FMRP-USP), Ribeirão Preto, SP, Brazil.; III MD, PhD. Cardiologist, Instituto do Coração (InCor), Hospital das Clínicas (HC), School of Medicine, Universidade de São Paulo (USP), São Paulo, and Hospital Municipal de Campo Limpo "Dr. Fernando Mauro Pires da Rocha", São Paulo, SP, Brazil.

**Keywords:** Histoplasmosis, Acquired immunodeficiency syndrome, Nasal septum, Histoplasma, Human immunodeficiency virus (HIV), Histoplasmose, Síndrome da imunodeficiência adquirida, Septo nasal, Histoplasma, Vírus da imunodeficiência humana (HIV)

## Abstract

**CONTEXT:**

Histoplasmosis is a fungal disease caused by inhaling spores of the fungus *Histoplasma capsulatum*. The spores can be found in soil contaminated with bird, bat or chicken feces. Histoplasmosis occurs worldwide and is one of the most common pulmonary and systemic mycoses.

**CASE REPORT:**

We report here the case of a 37-year-old man with acquired immune deficiency syndrome and histoplasmosis in the nasal septum, without pulmonary involvement, that evolved rapidly to disseminated infection, multiple organ failure and death.

## RESUMO

**CONTEXTO:**

Histoplasmose é uma doença fúngica causada pela inalação de esporos do fungo *Histoplasma capsulatum.* Os esporos podem ser encontrados no solo contaminado com fezes de aves, morcegos ou galinhas. Histoplasmose ocorre em todo o mundo e é uma das mais comuns micoses pulmonares e sistêmicas.

**RELATO DE CASO:**

Relatamos aqui o caso de um homem de 37 anos com síndrome da imunodeficiência adquirida e histoplasmose no septo nasal sem envolvimento pulmonar que evoluiu rapidamente para infecção disseminada, falência de múltiplos órgãos e morte.

## INTRODUCTION

Histoplasmosis is caused by a dimorphic fungus, *Histoplasma capsulatum*, that grows in the form of mycelia in soil (its natural habitat), in the presence of bird, bat and chicken feces.^1^ The infection starts after inhalation of microspores and it is generally asymptomatic or self-limited. Histoplasmosis may also result in variety of clinical manifestations. The most frequent is an asymptomatic or self-limited influenza-like respiratory infection.^1^ However, it also can be manifested as acute pulmonary histoplasmosis, chronic pulmonary histoplasmosis and disseminated histoplasmosis.^1^

We report here the case of a 37-year-old man with histoplasmosis in the nasal septum, without pulmonary involvement, that evolved very rapidly to disseminated infection, multiple organ failure and death.

## CASE REPORT

A 37-year-old man began to lose weight gradually after decreasing his food intake due to dysphagia in relation to both solid and liquid foods that began one month before admission. The decrease in food intake intensified over the last three days before his hospitalization because of an inflammatory and hemorrhagic process in his nose and fever. He did not have a cough, and had not had any previous similar episodes. Before hospitalization, the patient took several drugs without response. The patient’s medical history included moderate-risk sexual behavior (no condom) during the preceding four years and pulmonary tuberculosis five years earlier.

He presented difficulty in his vocal pronunciation associated with the growth of the hemorrhagic and inflammatory process in his nose. He was extremely thin (48 kg) and presented significant edema in the nasal area. He had an intermittent high temperature, which reached 40 °C, accompanied by chills. His pulse and blood pressure were normal, and heart and lung examinations showed normal results.

There were multiple non-painful lymph nodes of hard consistency in his neck. His nasal region showed phlogistic signs and skin fistulae, with bloody and purulent active secretion into the right ala, and other oronasal fistulae. The right nasal septum was very friable with tissue destruction.

The laboratory values were as follows: hemoglobin, 10.9 g/dl; hematocrit, 33.7%; platelets, 106; and white cell count, 3800/mm^3^, with 1% metamyelocytes, 2% less mature neutrophils (bands), 90% segmented neutrophils, 1% eosinophils, 5% lymphocytes and 1% monocytes. The aminotransferase levels were abnormal (aspartate, 774; and alanine, 136) and the albumin level was 2.1. The serological tests confirmed that the patient presented acquired immunodeficiency syndrome (AIDS). Chest radiographs were normal.

After the patient had been admitted to hospital, he was prescribed oxacillin, but this did not produce any response. This was then replaced by ceftriaxone, again without success. During hospitalization, computed tomography was performed, showing significant destruction of the nasal septum, with an image suggestive of severe nasal infection. Biopsies of the nasal septum lesion and lymph nodes were performed. At this point, metronidazole (400 mg, three times a day) was prescribed.

The following week, the patient presented intense vomiting, diarrhea, daily fever, nasal and oral hemorrhagic processes, anorexia and more weight loss. The patient began to show significant pancytopenia and two red blood cell packs were prescribed. The patient progressed badly and died a few days later.

The cervical lymph node that was biopsied did not reveal any histopathological alterations. From the biopsy material taken from the lesion in his nose and nasal septum, the fungus *Histoplasma capsulatum* was isolated.

## DISCUSSION

Both pulmonary involvement and disseminated disease are very common manifestations of histoplasmosis in immunocompromised patients presenting AIDS or chronic illness.^2-4^

Due to the small size of the fungal tissue (2-4 μm in diameter), and its similarity with other elements of yeast and other fungal structures, the definitive diagnosis of histoplasmosis is achieved by isolating the fungus in cultures.^1^ Thus, the diagnosis may take a long time to be established, and sometimes the treatment cannot be implemented in time.^1-4^ This was the case with our patient: only after his death was the definitive diagnosis of histoplasmosis made from the biopsy material that had been taken from his nasal septum during his hospitalization.

Because most of the clinical manifestations of histoplasmosis simulate tuberculosis, and the rapid clinical deterioration leads to multiple organ failure and death, histoplasmosis in our patient without pulmonary involvement was not diagnosed. Cases of histoplasmosis with skin lesions have been described among immunocompromised patients with AIDS.^5-22^ Although rare, patients with AIDS and skin lesions but without lung involvement have also been reported recently.^5-8^ Furthermore, an unusual orofacial case of manifestations of histoplasmosis in immunocompromised renal transplanted patient has also been described.^10^ Therefore, our objective in presenting this case was to draw attention to the diagnosis of histoplasmosis among patients with skin lesions alone, in the absence of pulmonary involvement, because such cases can also evolve very rapidly to systemic infection, multiple organ failure and death, especially among patients with AIDS.^2-7^ We performed a search in the relevant databases (Cochrane Database of Systematic Reviews; Embase Biomedical Answers; Literatura Latino-Americana e do Caribe em Ciências da Saúde; and United States National Library of Medicine and National Institutes of Health) for nasal septum and histoplasmosis, and the results are presented in Table 1.^9,10,23^

**Table 1.** Search in relevant databases for nasal septum and histoplasmosis

Databases	Search strategy	Results		
Cochrane	Nasal septum AND Histoplasmosis	0 article found	0 related article	–
Embase	Nasal septum AND Histoplasmosis	2 articles found^9,10^	2 related articles	2 case reports
Lilacs	Nasal septum AND Histoplasmosis	1 article found^23^	0 related article	1 case report
PubMed	Nasal septum AND Histoplasmosis	2 articles found^10,23^	1 related article	2 case reports

Cochrane = Cochrane Database of Systematic Reviews; Embase = Embase Biomedical Answers; Lilacs = Literatura Latino-Americana e do Caribe em Ciências da Saúde; PubMed = United States National Library of Medicine and National Institutes of Health.

## CONCLUSIONS

The special feature of this case report is that it presents the history of a 37-year-old man with AIDS and histoplasmosis in the nasal septum without pulmonary involvement who progressed very rapidly to disseminated infection, multiple organ failure and death. When there is no pulmonary involvement, the definitive diagnosis depends on isolation of the fungus in culture, which takes a long time. In these cases, in which the rapid progression of the disease prevents proper treatment, the diagnosis of histoplasmosis depends on a high degree of suspicion.
